# *In Vivo* Notch Signaling Blockade Induces Abnormal Spermatogenesis in the Mouse

**DOI:** 10.1371/journal.pone.0113365

**Published:** 2014-11-20

**Authors:** Daniel Murta, Marta Batista, Alexandre Trindade, Elisabete Silva, Domingos Henrique, António Duarte, Luís Lopes-da-Costa

**Affiliations:** 1 Reproduction and Development, Interdisciplinary Centre of Research in Animal Health (CIISA), Faculty of Veterinary Medicine, University of Lisbon, Lisbon, Portugal; 2 Gulbenkian Institute of Science, Oeiras, Portugal; 3 Institute of Molecular Medicine, Faculty of Medicine, University of Lisbon, Lisbon, Portugal; University of Nevada School of Medicine, United States of America

## Abstract

In a previous study we identified active Notch signaling in key cellular events occurring at adult spermatogenesis. In this study, we evaluated the function of Notch signaling in spermatogenesis through the effects of *in vivo* Notch blockade. Adult CD1 male mice were either submitted to a long term DAPT (?-secretase inhibitor) or vehicle treatment. Treatment duration was designed to attain one half the time (25 days) or the time (43 days) required to accomplish a complete cycle of spermatogenesis. Blockade of Notch signaling was depicted from decreased transcription of Notch effector genes. Notch signaling blockade disrupted the expression patterns of Notch components in the testis, induced male germ cell fate aberrations, and significantly increased germ cell apoptosis, mainly in the last stages of the spermatogenic cycle, and epididymis spermatozoa morphological defects. These effects were more pronounced following the 43 day than the 25 day DAPT treatment schedule. These results indicate a relevant regulatory role of Notch signaling in mammalian spermatogenesis.

## Introduction

Mammalian spermatogenesis involves continuous serial cellular proliferation and differentiation events, which occur in the complex cellular syncytium of the seminiferous tubules. These spatial-temporal cellular changes characterize the spermatogenic cycle, which, in the mouse, encompasses 12 stages [1], and require a finely tuned cellular signaling and a well-orchestrated gene expression. Deciphering the regulatory signaling behind these events could potentially lead to the development of new therapeutic strategies addressed to male infertility and contraception.

Notch is an evolutionarily conserved cell signaling pathway implicated in cell fate decisions in several tissues [2], [3]. In mammals, four receptors (Notch1–4) and five ligands (Dll1, Dll3, Dll4, Jagged1 and Jagged2) were described [3]. Notch signaling occurs following the coupling of the extracellular domain of receptors with ligands expressed on neighboring cells, which leads to cleavage of Notch intracellular domain (NICD) by a γ-secretase and its translocation to the nucleus. Here, it forms a complex with a transcriptional regulator RBP-jk and other co-regulators, inducing transcription of target effector genes [3]. From the limited set of Notch effector genes so far identified, the hairy/enhancer of split (Hes) genes are the most ubiquitous [4].

The role of Notch signaling in mammalian spermatogenesis is controversial. Several studies detected expression of Notch proteins in the testis of neonate and adult mice [5]–[9], or associated this pathway with male infertility [10]–[12]. However, two studies [13], [14], using genetically engineered mice, reported that the majority of Notch genes are not transcribed in the seminiferous tubules, and that Notch blockade in germ and Sertoli cells has no effects in the normal course of spermatogenesis. Recently, we identified active Notch signaling and described the expression patterns of Notch component and effector genes along mouse testis post-natal development and throughout the spermatogenic cycle [15]. Results prompted for a relevant role of Notch signaling in mammalian testis development and spermatogenesis.

The objective of this study was to evaluate the function of Notch signaling in spermatogenesis, through the effects of *in vivo* Notch blockade. Based on previous results, our hypothesis was that Notch signaling is a major regulator of germ cell fate decisions occurring during the spermatogenic cycle. To test this hypothesis we blocked *in vivo* Notch signaling using the γ-secretase inhibitor N-S-phenyl-glycine-t-butyl ester (DAPT). This methodology is an established approach to block Notch signaling *in vivo* and *in vitro*, and has been used in different scenarios [16]–[19]. Validating our hypothesis, blockade of Notch signaling induced abnormal spermatogenesis and spermatozoa defects. This phenotype indicates a relevant regulatory role of Notch signaling in mammalian spermatogenesis.

## Materials and Methods

### Animals

All experiments were conducted in accordance with the Portuguese legislation for the use of animals for experimental purposes (Decreto-Lei n° 129/92 and Portaria n° 1005/92, DR n° 245, série I-B, 4930-42) and with the European Union legislation (Directive n. 86/609/EEC, from the 24^th^ November 1986). Mice manipulation protocols were approved by the national regulatory agency (DGAV – Direção Geral de Alimentação e Veterinária) and the Institutional Animal Care and Use Committee (CEBEA – Comissão de Ética e Bem-Estar Animal). All authors are accredited as FELASA category C scientists or equivalent.

Outbred CD1 mice were chosen to introduce normal biological variability within the experiment. Mice were maintained in a 12-hour light/dark cycle, in ventilated cages with corn cob as bedding, and were given access to standard laboratory diet and water *ad libitum*. Mice health was monitored daily. Prior to the experiment, male mice were caged separately with used female bedding and, after four days, joined with a female until the production of a vaginal plug, to grant normal reproductive behavior and the presence of active spermatogenesis.

### Experimental design

Male mice (n = 24) with 3 months of age were randomly assigned to 4 groups: i) DAPT (Sigma-Aldrich, Inc., D5942) treatment during 25 days; ii) DAPT treatment during 43 days; iii) Control group (vehicle alone) during 25 days; and iv) Control group (vehicle alone) during 43 days. In adult mice, spermatogenesis (release of spermatozoa in the seminiferous tubule's lumen) takes 43 days [20]. We evaluated DAPT treatment effects at half the time (25 days) and at the time (43 days) required to accomplish a complete cycle of spermatogenesis. Side-effects of Notch blockade by γ-secretase inhibitors are time and dose dependent [21]. To reduce toxicity, a low dose regimen of 5 mg/Kg/day, 5 days per week was used [16]. DAPT powder was reconstituted in 100% ethanol (1 mg/mL) and stored in aliquots as stock solution at −20°C. Each day, fresh treatment solution was prepared, containing a mixture of 90% corn oil and 10% ethanol [16]. DAPT and vehicle alone were administered by oral gavage.

At the end of the experiment (25 and 43 days), during the last hour of dark, mice were euthanized through cervical dislocation under ketamine (15 mg/kg)/xylazine (1 mg/kg) anesthesia, followed by exsanguination. A blood sample was collected immediately before cervical dislocation and processed for plasma testosterone analysis. From each mouse, the testes were dissected free and processed for immunohistochemistry (IHC). One epididymis head was processed for RNA extraction, to evaluate the level of Notch signaling blockade through the transcription of main Notch effector genes (*Hes1*, *Nrarp*). This epididymis segment was elected based on its strong epithelial expression of Notch component and effector genes (data not shown), and its close anatomical proximity and functional relationship with the testis. One epididymis tail was used to collect spermatozoa to perform a sperm analysis.

### Quantitative transcription analysis (qRT-PCR)

Epididymis heads were immediately frozen in liquid nitrogen and stored at −80°C. RNA extraction, cDNA synthesis and mRNA transcription was performed as previously described [22]. Quantification of Notch effector genes *Hes1 and Nrarp* transcripts was done using selected primers (pair sequences available upon request). Transcription of gene *β2mg* was used as an endogenous control. Real-time PCR was performed in duplicate wells on StepOnePlus (Applied Biosystems, Foster City, CA, USA). All PCR reactions were carried out in 96-well optical reaction plates (Applied Biosystems, Warrington, UK) with 6.25 µl of 2x Power SYBR Green PCR Master Mix (Applied Biosystems, Warrington, UK), 2.5 ng of diluted cDNA and 80 nM of each primer in a total reaction volume of 12.5 µl.

### Histological evaluation and apoptosis assay

Paraffin sections were stained with haematoxylin. Spermatogenic cycle stages were identified as previously described [23]. Stages were grouped in two (I–II, III–IV, V–VI, VII–VIII, IX–X, XI–XII) to facilitate description.

Apoptosis was evaluated by the terminal deoxynucleotidyltransferase-mediated dUTP nick end labeling (TUNEL) assay, for *in situ* visualization of DNA fragmentation, according to manufacturer's instructions (Chemicon, Millipore). The percentage of apoptotic cells was determined for each cell type and spermatogenic cycle stage. This evaluation was performed on a total of 30 seminiferous tubules per animal.

### Immunohistochemistry (IHC)

The spatial expression patterns of Notch components were determined following IHC, according to a method previously described [24]. Staining was evaluated in the entire slice. Testis expression patterns were established following the evaluation of a minimum of 36 slices (3 slices/testis x 2 testis x 6 animals) for each Notch component (plus 12 twin-slides with cell marker) in each of the four experimental groups.

The anti-3β-HSD antibody was used to identify Leydig cells and the anti-DAZL antibody was used to identify germ cells. The antibodies against Notch components and effectors were previously validated by others in the mouse (anti-Notch1 [25], anti-Notch3 [26], anti-Dll1 [27], anti-Dll4 [27], anti-Jagged1 [28]), and rat species (anti-Notch2 [17]). The antigen retrieval step was performed in citrate buffer (10mM, pH 6.0), except for the anti-Notch1 antibody (Tris-EDTA, pH 9.0). Blocking was performed in PBS with 2% bovine serum albumin (Sigma-Aldrich, Inc.) for 1 hour at room temperature. Sections were incubated overnight at 4°C with each primary antibody: Notch1 (Ab27526, Abcam), diluted 1∶100; Notch2 (Ab8926, Abcam), diluted 1∶100; Notch3 (Ab23426, Abcam), diluted 1∶160; Dll1 (Ab10554, Abcam), diluted 1∶100; Dll4 (Ab7280; Abcam), diluted 1∶200; Jagged1 (SC-8303, Santa Cruz Biotechnology), diluted 1∶50; 3β-HSD (SC-30820, Santa Cruz Biotechnology), diluted 1∶300, and DAZL (Ab34139; Abcam), diluted 1∶250. Negative controls used the polyclonal rabbit IgG (Ab27478, Abcam), diluted 1∶100 and, for the 3β-HSD antibody, the goat control IgG (Ab37373, Abcam), diluted 1∶300. All primary antibodies were diluted in blocking solution. The peroxidase conjugated monoclonal mouse anti-goat/sheep IgG antibody (A9452, Sigma-Aldrich, Inc.), diluted 1∶100, was used as secondary antibody for the 3β-HSD antibody, and the peroxidase conjugated goat anti-rabbit IgG polyclonal antibody (Dako 410972), diluted 1∶100, was used as secondary antibody for the remaining primary antibodies.

### Epididymis sperm analysis

Epididymis tails were placed in 200 µl of Human Tubarian Fluid (HTF) medium at 37°C, and sliced to allow recovery of luminal fluid. After 5 minutes, tissue was removed from medium, and samples of the sperm suspension taken for analysis. Sperm concentration was evaluated in a Neubauer chamber. Sperm morphology was evaluated in eosin-nigrosin stained slides.

### Measurement of plasma testosterone concentrations

Total plasma testosterone was assayed without extraction by a solid-phase, competitive chemiluminescent enzyme immunoassay (Immulite 1000, Siemens Healthcare Diagnostics, Lda., Amadora, Portugal), using a commercial kit (Immulite 1000 Total Testosterone kit, Siemens). The inter-assay coefficients of variation were 8.9% and 9.7% for two different controls used in the assay (for concentrations of 279 and 735 ngdL−1, respectively; Multivalent Control Module, Siemens Healthcare Diagnostics). The intra-assay coefficient of variation was 5.1%.

### Statistical analysis

Data analysis was performed with the SPSS version 19.0 statistic software. Comparisons between groups used the Student's t-test, One-way ANOVA or Brown-Forsythe test, followed by Bonferroni or Tamhane's T2 post-hoc tests (assuming homogeneity of variances or not, respectively). Significance was determined at the 5% confidence level (*p*<0.05).

## Results

### DAPT treatment decreased transcription of Notch effector genes *Hes1* and *Nrarp*

In DAPT treated mice, transcription levels in the epididymis head decreased approximately 30-10% for genes *Hes1* and *Nrarp*, compared with control mice (Figure S1).

### DAPT treatment disrupted expression patterns of Notch components in the testis

**Figure 1:**
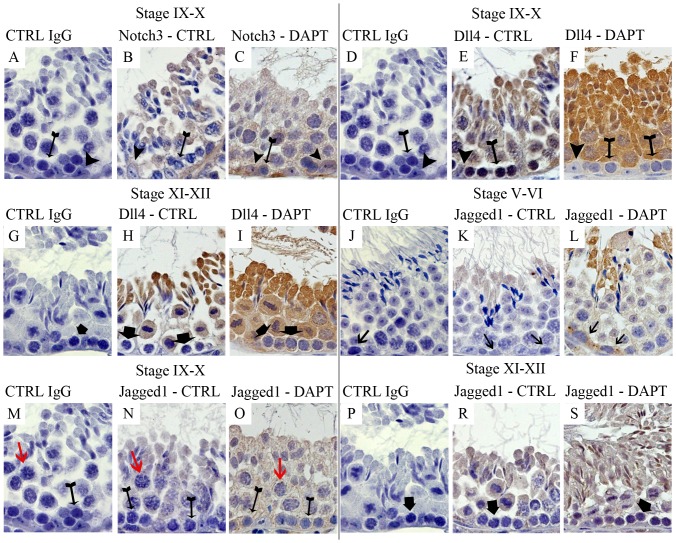
Disruption of expression patterns of Notch components along the spermatogenic cycle following DAPT treatment. Positive staining in brown, counterstaining with haematoxylin (400x magnification). Arrow heads point Sertoli cells. Tailed arrows point leptotene spermatocytes. Bold arrows point zygotene spermatocytes. Arrows point spermatogonia. Red arrows point pachytene spermatocytes. Control (CTRL) slides used rabbit IgG (A, D, G, J, M, P). Notch3 shows ectopic expression in Sertoli cells and in leptotene spermatocytes at stages IX–X. (B and C). Dll4 stops being expressed in leptotene spermatocytes and Sertoli cells at stage IX–X (E and F), and shows ectopic expression in zygotene spermatocytes at stage XI–XII (H and I). Jagged1 shows ectopic expression in spermatogonia at stages V–VI (K and L); in leptotene and pachytene spermatocytes at stages IX–X, (N, O); and in zygotene spermatocytes at stages XI–XII (R and S).

Disruption of the normal expression patterns was already foreseeable after 25 days of treatment (data not shown), but became evident after 43 days of DAPT treatment. Figure 1 show the main changes in expression detected in DAPT treated mice, which are also schematically illustrated in Figures S2 and S3. Notch1, Notch2 and Dll1 did not change their expression patterns. Notch3 showed ectopic expression in Sertoli cells at stages VI-IX and in leptotene and zygotene spermatocytes at stages IX–XII (Figure 1, B and C). Dll4 stopped being expressed in leptotene spermatocytes and Sertoli cells at stage IX–X (E and F), and showed ectopic expression in pachytene spermatocytes at stage I–IV and in zygotene spermatocytes at stage XI–XII (H and I). Jagged1 showed the most extensive changes in the expression pattern following DAPT treatment. Ectopic expression was observed in spermatogonia at stages II–VII (K and L), in round spermatids at stage VII–VIII, in leptotene and pachytene spermatocytes at stages IX–X (N and O), and in zygotene spermatocytes at stages XI–XII (R and S). Additionally, Jagged1 stopped being expressed in diplotene spermatocytes and germ cells finalizing meiosis at stages XI–XII (R and S).

**Figure 2:**
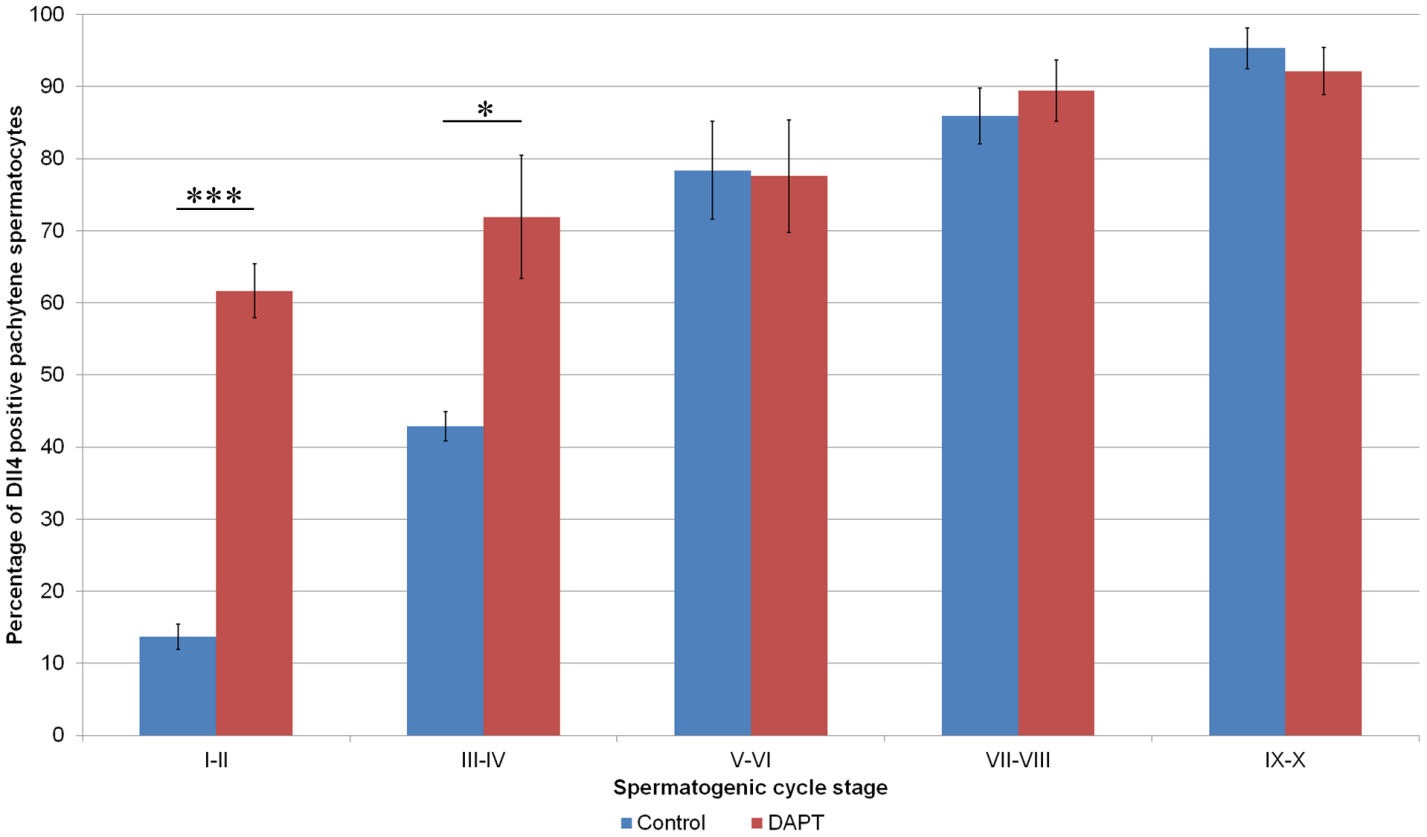
DAPT treatment significantly changes the proportion of pachytene spermatocytes expressing Dll4. (_*_) *p*<0.05; (_***_) *p*<0.001

Figure 2 shows the percentage of pachytene spermatocytes expressing Dll4 along the spermatogenic cycle. As can be depicted, DAPT treatment significantly advanced the expression of this ligand in the spermatogenic cycle. The expression of other Notch components in pachytene spermatocytes was not significantly changed.

**Figure 3:**
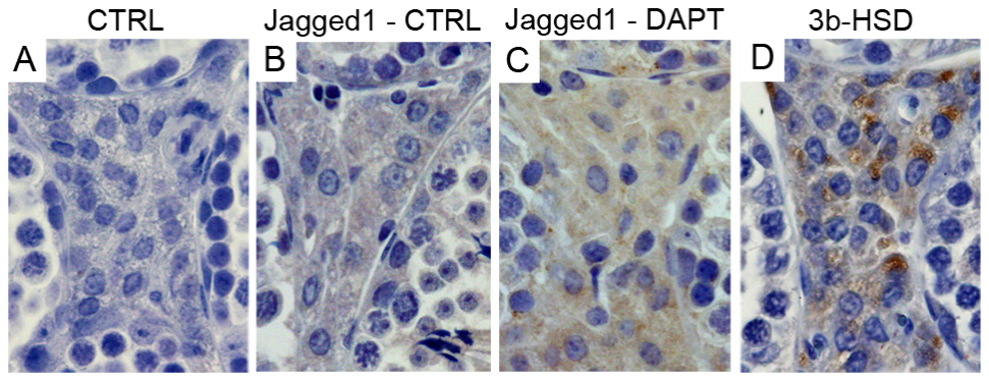
DAPT treatment disrupts Jagged1 expression in testis interstitial Leydig cells. Positive staining in brown, counterstaining with haematoxylin (400x magnification). Control (CTRL) slides used rabbit IgG (A). Jagged1 is not expressed in Leydig cells of control mice (B), but becomes expressed following DAPT treatment (C). Leydig cells are co-localized in twin slides with marker 3β-HSD (D).

Following DAPT treatment, interstitial Leydig cells gained expression of Jagged1 (Figure 3), whereas all other Notch components did not change their expression pattern.

### DAPT treatment induced germ cell fate aberrations and germ cell abnormal morphology

**Figure 4:**
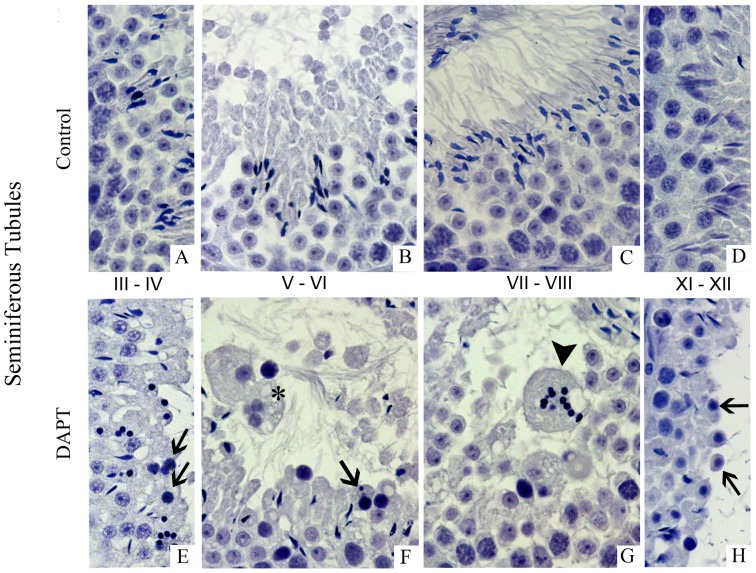
DAPT treatment induces formation of abnormal germ cells. Haematoxylin staining (400x magnification). Arrows mark spermatids that fail to elongate. Arrow heads mark germ cells with several small nucleus/nuclear fragments. Asterisk marks giant multinucleated germ cell. Seminiferous tubules from control mice (A–D). Elongating spermatids appear with several round small nuclei (E, H). Giant multinucleated germ cells (F). Enlarged germ cells with vacuolated cytoplasm and several nuclear fragments (G). Round spermatids fail to elongate, become round, pyknotic, and are released in the lumen (F, H).

**Figure 5:**
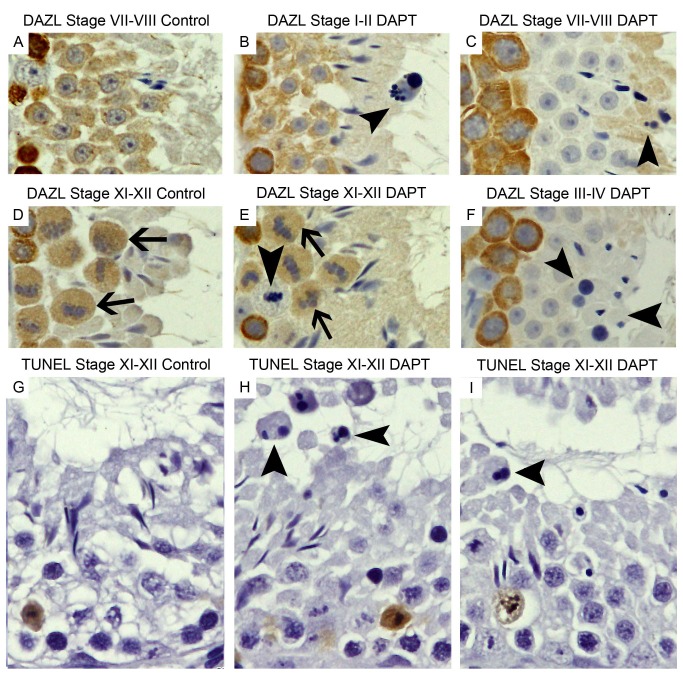
Abnormal germ cells loose identity. Positive staining in brown, counterstaining with haematoxylin (400x magnification). Germ cells of control mice stain with marker DAZL (A and D). Most abnormal germ cells of DAPT treated mice do not stain with marker DAZL (B, C, E and F). TUNEL staining of seminiferous tubules of control (G) and DAPT treated (H and I) mice. Abnormal germ cells are TUNEL negative. Arrow heads point abnormal germ cells. Arrows point normal dividing germ cells.

**Figure 6:**
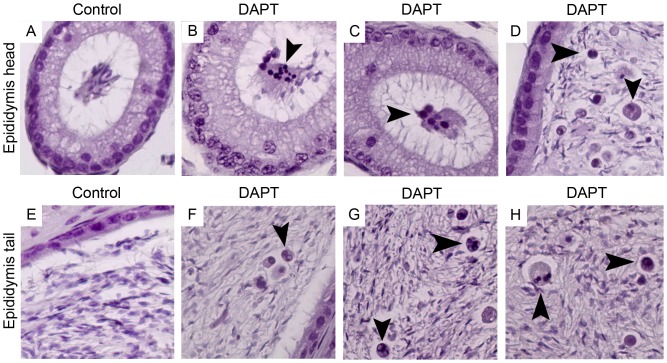
Abnormal germ cells are released into the epididymis lumen. Staining with haematoxylin (400x magnification). Tissue sections from epididymis head and tail lumen of control (A and E) and DAPT treated (B–D and F–H) mice. DAPT treated mice show abnormal germ cells in the epididymis lumen. Arrow heads point abnormal germ cells.

Figure 4 shows the main effects of DAPT treatment on germ cell fate and morphology. Abnormal germ cell fate and morphology were observed in almost all seminiferous tubules, and although the majority of germ cells proceeded in differentiation, this largely leaded to the production of morphologically abnormal cells. Round spermatids failed to elongate, became round, pyknotic and were released into the lumen (E, H), sometimes displaying a small nucleus (E). Other germ cells failed to enter a normal cell division. These cells became enlarged, with vacuolated cytoplasm and several nuclear fragments (F and G). These anomalous cells expressed all Notch components, were not marked by DAZL (Figure 5 A–F), were TUNEL negative (Figure 5 G–I), and were released in the seminiferous tubule lumen, being also identified in the epididymis lumen (Figure 6).

### DAPT treatment induced increased apoptosis in germ cells

**Figure 7:**
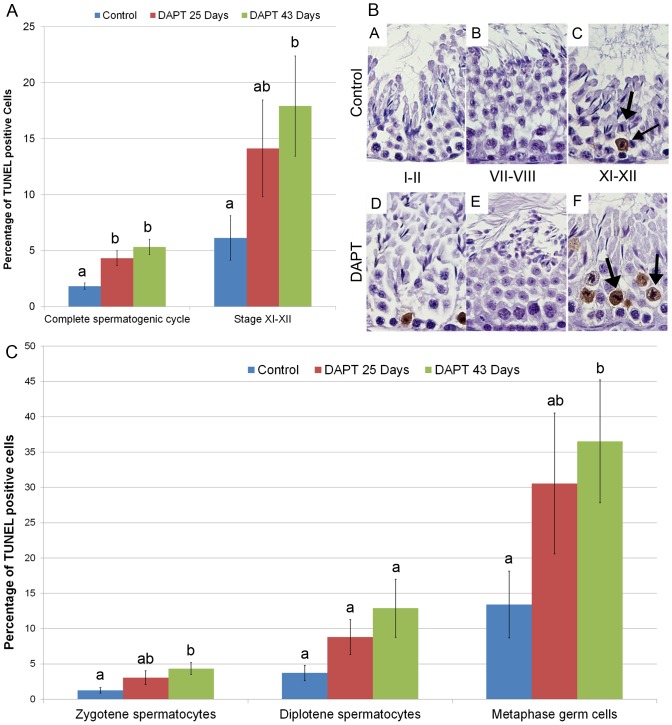
DAPT treatment significantly increases apoptosis during spermatogenesis. **A**– Relative frequency of apoptotic germ cells at all spermatogenic cycle stages and at stages XI–XII. Error bars represent the standard error of the mean (SEM). Columns with different superscript differ significantly. ab, *p*<0.05. **B** – Apoptosis TUNEL evaluation. TUNEL positive staining in brown, counterstaining with haematoxylin (400x magnification). Arrows mark cells at last steps of meiosis. Apoptosis in seminiferous tubules of control (A–C) and DAPT treated (D–F) mice. Cells at last steps of meiosis are more affected (C, F). **C** – Relative frequency of apoptotic germ cell types at the most affected spermatogenic stage (XI–XII). Error bars represent the standard error of the mean (SEM). Columns with different superscript differ significantly: ab, *p*<0.05.

As shown in Figure 7A, compared to control groups, DAPT treatment induced a significantly higher rate of apoptosis in germ cells. However, the apoptotic rate in germ cells was not significantly different following the two DAPT treatment lengths. Apoptosis was mainly observed at the XI–XII stage, and overall differences in the apoptosis rate observed between DAPT treatment and control groups, arose mainly from differences observed at this cycle stage. Zygotene spermatocytes and germ cells undergoing last steps of meiotic divisions were the most affected by DAPT treatment (Figure 7 B and C).

### DAPT treatment increased morphologic defects in epididymis spermatozoa

**Figure 8:**
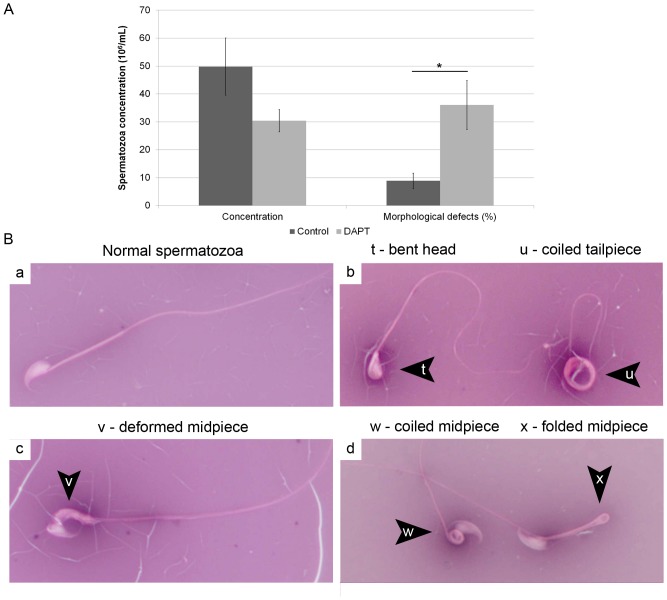
DAPT treatment significantly increases epididymis spermatozoa morphologic defects. **A**– Epididymis tail spermatozoa concentration (number of spermatozoa per mL of collection medium) and morphology evaluation. (_*_) *p*<0.05 **B** – Main spermatozoa morphologic defects found in DAPT treated mice, eosin-nigrosin stain (400x magnification). Normal spermatozoa (a), morphological defects (b–d).

As shown in Figure 8A, although sperm concentration was not significantly affected by DAPT treatment, as expected from testis histology results, the proportion of spermatozoa with morphological defects was significantly higher in DAPT treated mice than in control mice. The main morphological defects were primary anomalies of the sperm head and midpiece, including bent, thin and club-shaped heads, coiled, folded and deformed midpieces, and coiled tailpieces (Figure 8B).

### Plasma testosterone concentrations were not affected by DAPT treatment

Plasma testosterone concentrations of DAPT treated and control mice were similar (Figure S4).

## Discussion

We recently described the transcription and expression patterns of Notch component and effector genes in mouse testis post-natal development and along the adult spermatogenic cycle [15]. Results indicated that Notch signaling is active, and prompted for a relevant regulatory role of Notch signaling in testis development and during spermatogenesis. This conducted to the phenotypic evaluation of *in vivo* blockade of Notch signaling using the γ-secretase inhibitor DAPT. To evaluate the level of Notch signaling blockade, transcription of main Notch effector genes was analyzed. DAPT decreased transcription of Notch effector genes, which evidences that treatment attained the expected biological effect. However, the efficacy of treatment was incomplete, probably due to the low drug dosage used. Therefore, results here discussed have to be addressed assuming that most of Notch signaling is still potentially active, and that a more efficacious blockade could induce a more representative picture of the role of Notch signaling in spermatogenesis.

*In vivo* Notch blockade disrupted expression patterns of Notch components (Notch3, Dll4 and Jagged1). Enhanced expression of Notch3 may be due to a decrease in the activation of other receptors. This can also lead to the increase in available ligands and justify the enhanced expression of Dll4 and Jagged1, as a compensatory effect. However, as germ cell fate and identity are probably the result of unique expression combinations of receptors and ligands [15], ectopic expression of Notch components may induce disturbances in these events, and induce abnormal progression of spermatogenesis, as observed in this study.

*In vivo* Notch blockade induced the formation of morphologically abnormal germ cells, including elongated spermatids with a round nucleus and multinucleated vacuolated giant cells. The ectoplasmic specialization is the elongated spermatid anchoring system, which maintains adherence to Sertoli cells, and confers cell orientation and polarity within the seminiferous epithelium [29]. This spermatid anchoring system is mainly composed by Par complex proteins [29]. Notch signaling is associated to cell polarity decisions in tissues involving Par complex proteins, such as the neural epithelium [30], [31]. We have recently reported expression of Notch components in the elongated spermatid anchoring system, and nuclear detection of Hes5 in Sertoli cells and in elongating spermatids [15]. Notch blockade induced abnormal spermatid elongation and its premature release into the lumen, and the presence of abnormal cells in the lumen of seminiferous tubules and epididymis. This indicates a regulatory role of Notch signaling in spermatid's elongation and maintenance of the elongated spermatid anchoring system. Notch pathway was associated with cell division [31], [32]. The formation of multinucleated cells and the increased apoptosis of germ cells undergoing the final steps of the meiotic divisions, following Notch blockade, indicate that Notch signaling is involved in the regulation of germ cell meiosis.

*In vivo* Notch blockade significantly increased the rate of apoptosis in germ cells, mainly at stage XI–XII of the spermatogenic cycle. Zygotene spermatocytes were the germ cells most affected by apoptosis. Interestingly, these germ cells do not normally express Notch components [15], but exhibited ectopic expression of Notch3, Dll4 and Jagged1 following DAPT treatment. Apoptosis in these germ cells may follow loss of identity due to blockade of Notch signaling. However, abnormal germ cells stopped expressing marker DAZL (which indicates loss of germ cell identity), but did not become apoptotic.

*In vivo* Notch blockade disrupted the expression patterns of Notch components in Leydig interstitial cells, inducing ectopic expression of Jagged1. However, no significant effect on plasma testosterone concentrations was evident.

*In vivo* Notch blockade significantly increased the proportion of epididymis spermatozoa with primary morphological defects. This feature probably arises from disturbances in spermatogenesis, as discussed above. The increase in sperm morphological defects is associated with reduced fertility [33]. Although experimental mice were not allowed to breed following DAPT treatment, the obtained effect could lead to a reduced fertility; however, the putative effects of abnormal Notch signaling on fertility are probably related with the level of Notch pathway activity. Notch deregulation in the testis was associated with male fertility problems [10]–[12], [34], and potentially linked with maintenance and growth of testicular germ cell tumors [8], [35].

In conclusion, *in vivo* Notch blockade disrupted expression patterns of Notch components in the testis, induced germ cell fate aberrations, morphological abnormalities and apoptosis, and increased morphological defects in epididymis spermatozoa. This indicates that Notch signaling has a major regulatory role in spermatogenesis, namely in germ cell fate and identity, meiosis and differentiation of spermatids. This turns the Notch pathway into an attractive fertility therapeutic target.

## Supporting Information

Figure S1 **DAPT decreases transcription of downstream Notch effector genes** ***Hes1 and Nrarp*** **.** Comparison between a representative animal of each group (Control and DAPT treatment).
(TIF)

Figure S2 **Schematic illustration of expression patterns of Notch pathway receptors along the spermatogenic cycle: comparison between control and DAPT treated mice.** Draw-scheme representing stages (I–XII) of the spermatogenic cycle. Spermatogonia (A, In, B); spermatocytes (Pl- preleptotene, L- leptotene, Z- zygotene, P- pachytene, D- diakinesis, Mi- meiotic division); round spermatids (1–8); elongated spermatids (9–16). Spatial localization of expression of Notch receptors drawn in different colors, according to legend. Drawing adapted from Hess and Franca (2008).
(TIF)

Figure S3 **Schematic illustration of expression patterns of Notch pathway ligands along the spermatogenic cycle: comparison between control and DAPT treated mice.** Draw-scheme representing stages (I–XII) of the spermatogenic cycle. Spermatogonia (A, In, B); spermatocytes (Pl- preleptotene, L- leptotene, Z- zygotene, P- pachytene, D- diakinesis, Mi- meiotic division); round spermatids (1–8); elongated spermatids (9–16). Spatial localization of expression of Notch ligands drawn in different colors, according to legend. Drawing adapted from Hess and Franca (2008).
(TIF)

Figure S4 **Plasma testosterone concentrations of control and DAPT treated mice.**
(TIF)
