# Supplementary figures and images for: In Vivo Notch Signaling Blockade Induces Abnormal Spermatogenesis in the Mouse
Source: PLoS One. 2014 Nov 20;9(11):e113365. doi: 10.1371/journal.pone.0113365 (PMC4239051; doi:10.1371/journal.pone.0113365)

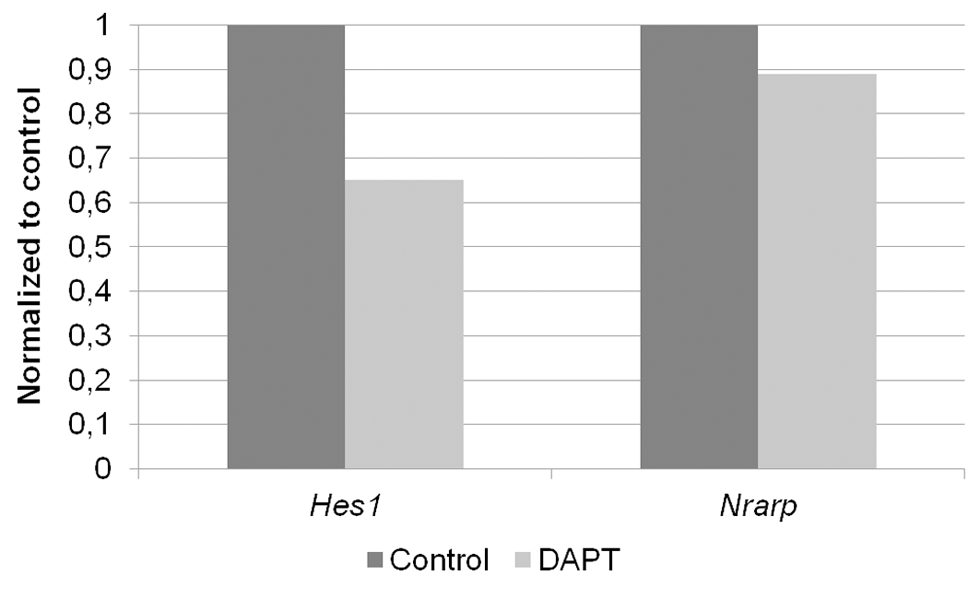

Supplement: Figure S1 — DAPT decreases transcription of downstream Notch effector genes Hes1 and Nrarp . Comparison between a representative animal of each group (Control and DAPT treatment). (TIF) [file pone.0113365.s001.tif]

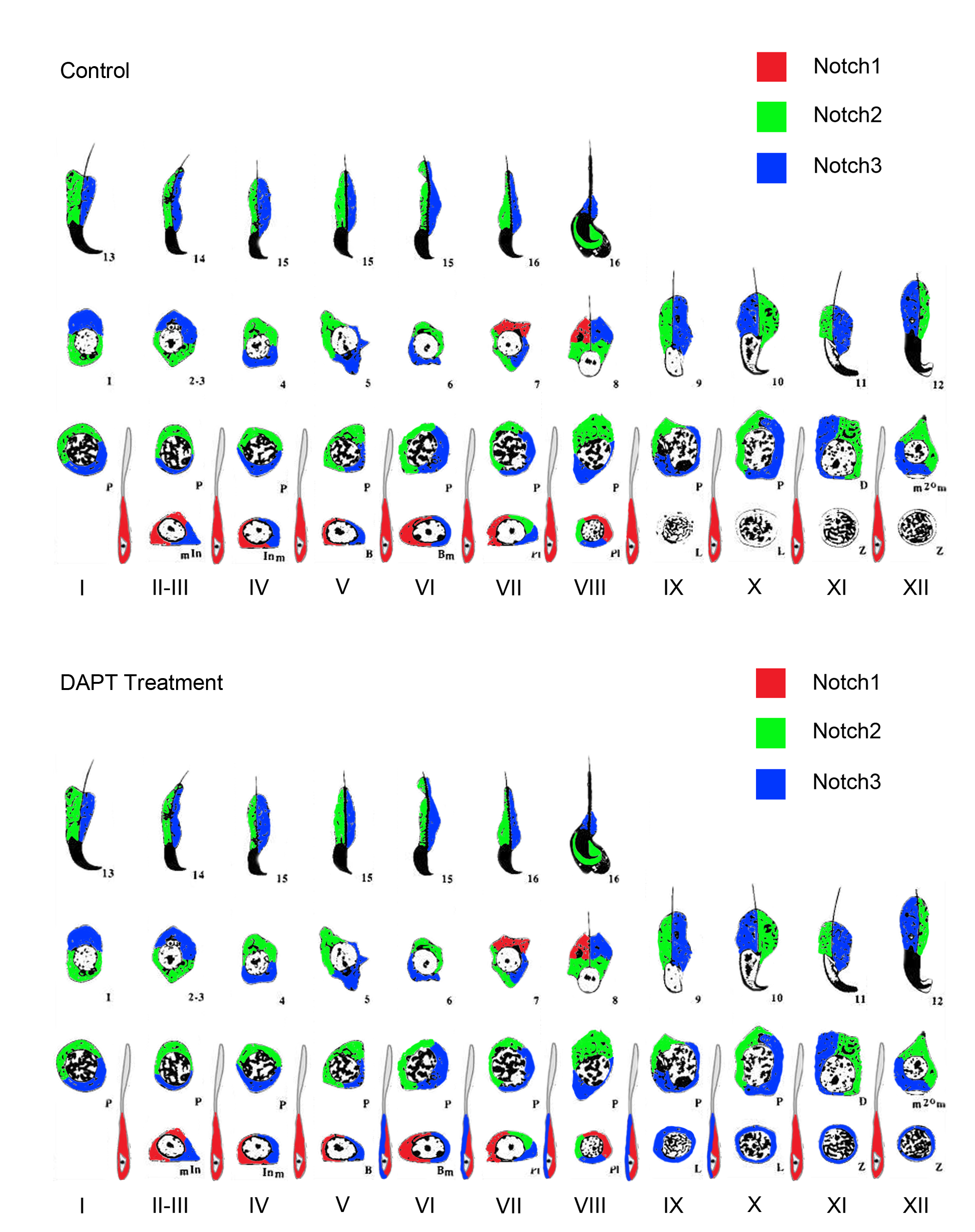

Supplement: Figure S2 — Schematic illustration of expression patterns of Notch pathway receptors along the spermatogenic cycle: comparison between control and DAPT treated mice. Draw-scheme representing stages (I–XII) of the spermatogenic cycle. Spermatogonia (A, In, B); spermatocytes (Pl- preleptotene, L- leptotene, Z- zygotene, P- pachytene, D- diakinesis, Mi- meiotic division); round spermatids (1–8); elongated spermatids (9–16). Spatial localization of expression of Notch receptors drawn in different colors, according to legend. Drawing adapted from Hess and Franca (2008). (TIF) [file pone.0113365.s002.tif]

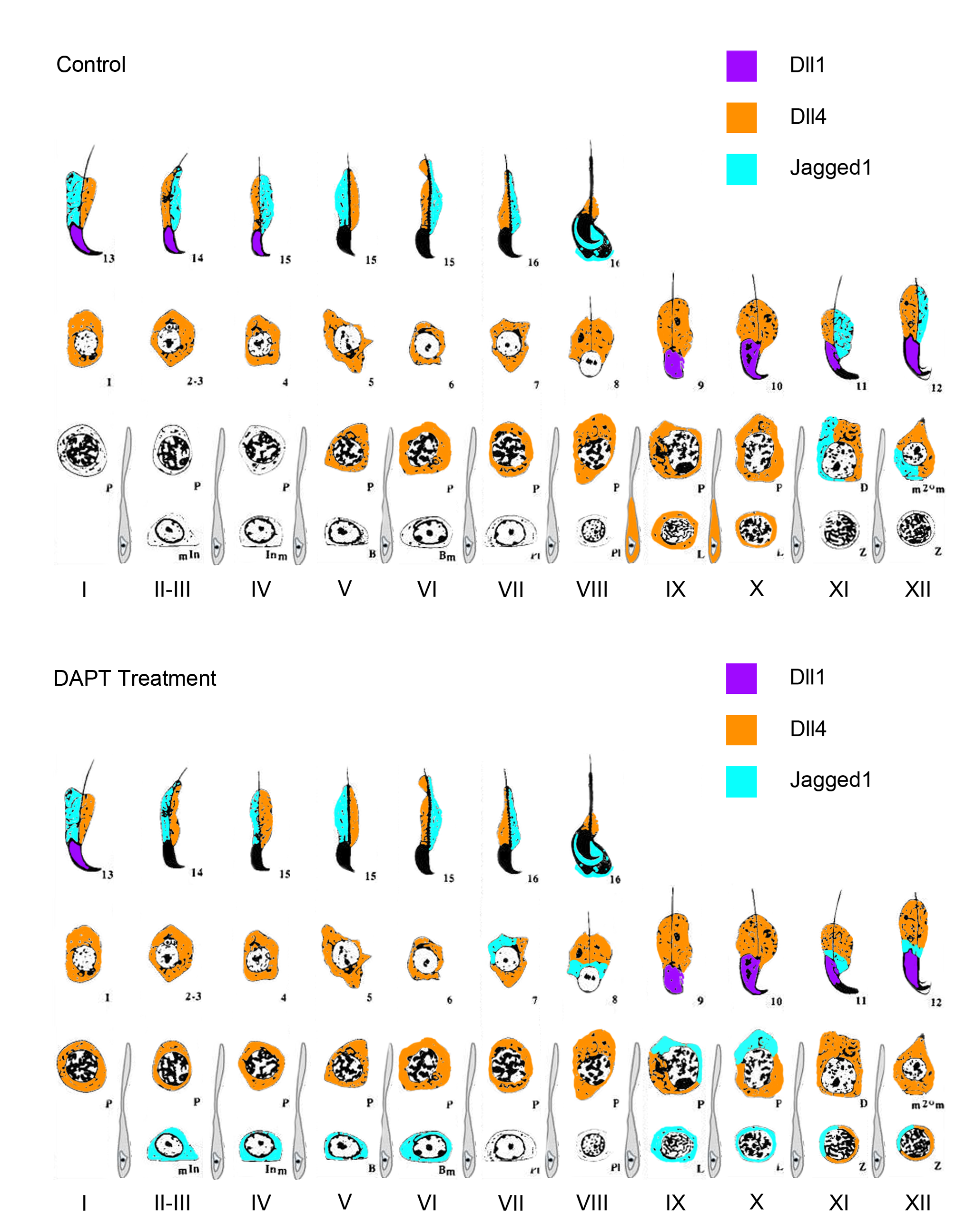

Supplement: Figure S3 — Schematic illustration of expression patterns of Notch pathway ligands along the spermatogenic cycle: comparison between control and DAPT treated mice. Draw-scheme representing stages (I–XII) of the spermatogenic cycle. Spermatogonia (A, In, B); spermatocytes (Pl- preleptotene, L- leptotene, Z- zygotene, P- pachytene, D- diakinesis, Mi- meiotic division); round spermatids (1–8); elongated spermatids (9–16). Spatial localization of expression of Notch ligands drawn in different colors, according to legend. Drawing adapted from Hess and Franca (2008). (TIF) [file pone.0113365.s003.tif]

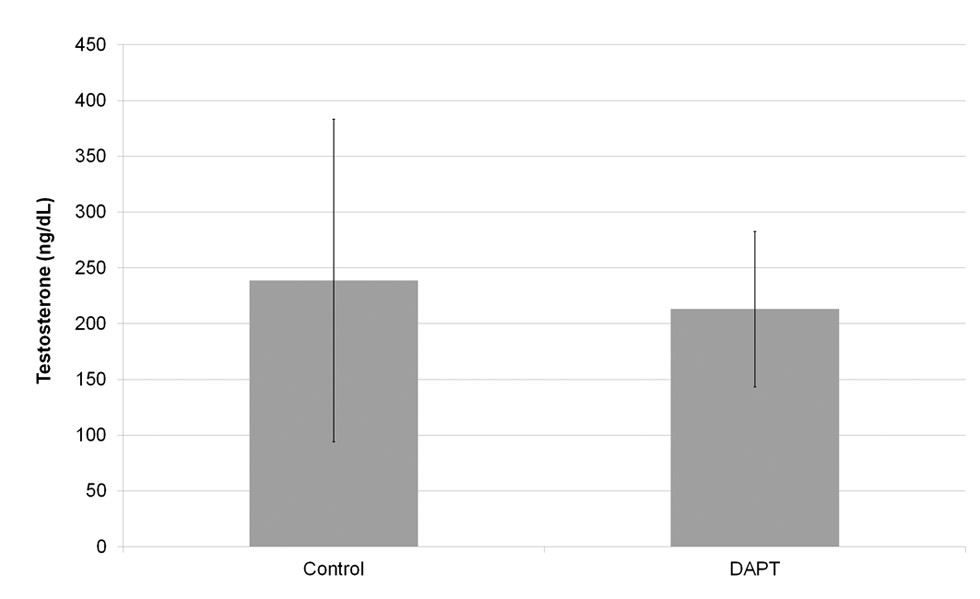

Supplement: Figure S4 — Plasma testosterone concentrations of control and DAPT treated mice. (TIF) [file pone.0113365.s004.tif]
